# Expression and Function of Toll Pathway Components in the Early Development of the Wasp *Nasonia vitripennis*

**DOI:** 10.3390/jdb10010007

**Published:** 2022-01-26

**Authors:** Daniel Pers, Thomas Buchta, Orhan Özüak, Siegfried Roth, Jeremy A. Lynch

**Affiliations:** 1 Department of Biological Sciences, University of Illinois at Chicago, Chicago, IL 60607, USA; dpers88@gmail.com; 2 Department of Biochemistry, Vanderbilt University, Nashville, TN 37232, USA; 3 Department of Developmental Biology, University of Cologne, 50674 Cologne, Germany; thomas_buchta@yahoo.de (T.B.); o.oezueak@gmail.com (O.Ö.); siegfried.roth@uni-koeln.de (S.R.)

**Keywords:** *Nasonia*, toll, embryonic patterning, dorsal-ventral

## Abstract

The Toll signaling pathway is the main source of embryonic DV polarity in the fly *Drosophila melanogaster.* This pathway appears to have been co-opted from an ancestral innate immunity system within the insects and has been deployed in different ways among insect taxa. Here we report the expression and function of homologs of the important components of the *D. melanogaster* Toll pathway in the wasp *Nasonia vitripennis.* We found homologs for all the components; many components had one or more additional paralogs in the wasp relative the fly. We also found significant deviations in expression patterns of *N. vitripennis* homologs. Finally, we provide some preliminary functional analyses of the *N. vitripennis* homologs, where we find a mixture of conservation and divergence of function.

## 1. Introduction

The parasitoid wasp, *Nasonia vitripennis*, is an emerging genetic model system that has experienced much growth in both its genetic toolkit and its applications over the last decade [1,2,3,4,5]. Embryonic patterning is a topic of particular interest in *N. vitripennis*, as it undergoes a rapid mode of long germ embryogenesis, that is very similar in many ways to that of the well-described model system *D. melanogaster*, but with some derived features reflecting the independent evolutionary derivation of this mode of embryogenesis [6,7].

While the fundamental similarity of the wasp and fly early embryonic patterning output was originally observed for anterior–posterior patterning [4,7], the similarity was later found to extend to the orthogonal dorsal–ventral (DV) axis [8]. For DV patterning, it was found that the expression of markers of cell fate was highly conserved between the fly and wasp just prior to the onset of gastrulation. However, the way the patterns arise earlier in development and how the tissues behave after gastrulation were quite distinct [8,9].

Relevant here are the DV patterning processes leading up to gastrulation. In *D. melanogaster* the Toll signaling pathway serves to polarize the axis and to provide positional information to the ventral half of the embryo [10,11,12,13,14,15,16,17,18,19,20,21]. It also controls the expression of several components of the BMP signaling pathway, leading to a BMP gradient emanating from the dorsal pole of the embryo that patterns the dorsal half of the embryo [22,23,24,25,26,27,28,29,30,31,32,33,34,35]. Loss of Toll signaling leads to complete loss of DV polarity, while loss of BMP has less extensive effects.

In *N. vitripennis*, the opposite pattern prevails. Loss of BMP signaling leads to loss of DV polarity, while loss of Toll signaling leads only to the loss of the ventral-most fates [36]. Previous examination of the BMP signaling components in *N. vitripennis* revealed some significant divergences from the *D. melanogaster* pathway [37], and the significance of these differences to the divergent function of the wasp BMP pathway are currently under investigation. Here, we present our analysis of the Toll signaling pathway in *N. vitripennis* and its significance for patterning the wasp embryo.

**Figure 1:**
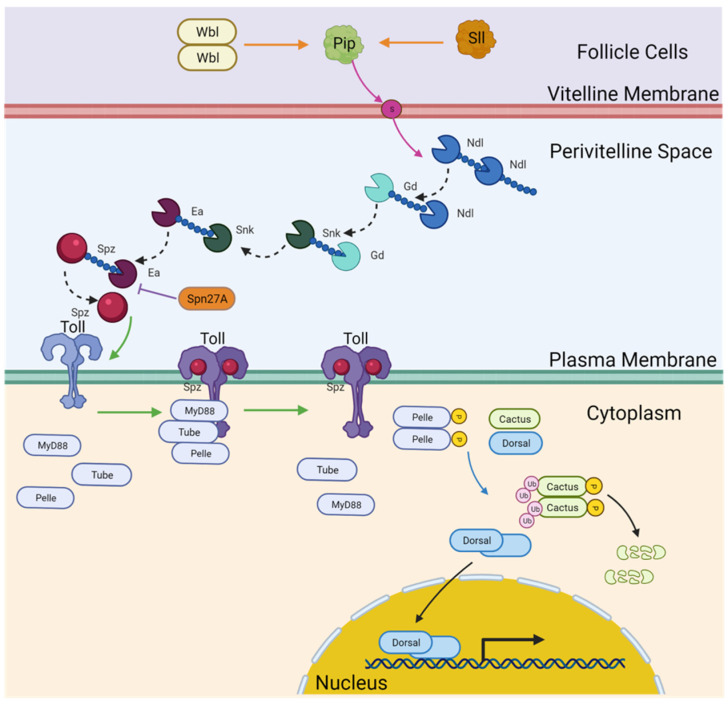
Summary of the components of the Drosophila Toll pathway used for comparison here. Created with *BioRender.com*.

In *D. melanogaster*, the Toll–Dorsal Pathway is known to be activated by a complex upstream network, signaling back and forth between the follicle cells, perivitelline space, and the egg (Figure 1). This signaling network is set into motion by asymmetric localization of the nucleus within the oocyte [38,39,40]. This asymmetry leads to activation of EGF signaling in follicle cells that will define the future dorsal half of the eggshell [38,39,40,41,42]. EGF signaling represses transcription of the sulfotransferase encoding gene *pipe* in the dorsal region, thus restricting it to the ventral half of the egg [38,39,40,41,43,44,45]. Pipe (Pip), aided by the ER resident protein Windbeutel (Wbl) and the sulfur transporter Slalom (Sll), sulfates multiple vitelline membrane proteins, which provides an asymmetric signal to proteases acting in the perivitelline space [45,46,47,48,49,50,51]. The protease cleavage cascade of Gastrulation Defective (Gd), Snake (Snk), and Easter (Ea), facilitated by Nudel (Ndl) protease activity, ends with cleavage of the ligand Spätzle (Spz) [21]. A Spz cleavage product forms a ventral-to-dorsal gradient and can bind the Toll receptor on the embryonic plasma membrane [52,53,54,55,56,57,58,59,60]. Once Spz binds to the Toll receptor, Toll internalizes and frees Pelle from an intracellular complex, including Toll, Pelle (Pll), Tube (Tub), and Myd88 [10,11,12,13,61,62,63,64,65]. Pelle can then phosphorylate Cactus (Cact), causing dissociation from Dorsal (Dl) [15,66,67,68,69,70,71]. Free Dl can then translocate to the nucleus, where it acts as a transcription factor, activating and repressing hundreds of downstream targets responsible for establishing cell fate identities in the embryo [16,18,19,20,22,23,72,73,74].

The goal of this work is to identify and initially characterize the components of the *N. vitripennis* Toll signaling pathway in detail in order to fill in our gaps of knowledge and, together with previous work, provide a more complete picture of the mechanism of this dynamic DV patterning system in the wasp. A candidate gene approach was used to find wasp orthologs to all known fly components and compare their expression through in situ hybridization. In addition, preliminary functional studies were conducted in *N. vitripennis* on major pathway components in order to elucidate the inner working of the pathway.

## 2. Materials and Methods

### 2.1. Discovery of Toll Pathway Orthologs

Protein sequences of *Drosophila melanogaster* Toll Pathway components were obtained from the NCBI “Protein” database. Potential *N. vitripennis* orthologs were obtained by submitting the corresponding *D. melanogaster* sequence into the query of the “blastp suite” of the NCBI Basic Local Alignment Search Tool (BLAST) [75]. Search settings were limited by “Database” (non-redundant protein sequences (nr)) and “Organism” (Nasonia vitripennis (taxid:7425)). “BLAST Results” were analyzed by reciprocal BLASTing the top hit under the “Sequences producing significant alignments” header.

The sequence of the top hit was entered into a new “blastp suite” query and BLASTed against the *D. melanogaster* database (“Organism”: Drosophila melanogaster (taxid:7227)). If the original *D. melanogaster* sequence entered was the top hit of this second BLAST, then the *N. vitripennis* sequence found was considered an ortholog.

Since there is always the possibility of lineage-specific duplications, we repeated the reciprocal blast with the next best hit, until the *N. vitripennis* gene returned a *D. melanogaster* sequence other than the original query. All *N. vitripennis* sequences that returned the original query (or its known paralog, if any) were also saved and were considered potential orthologs. These potential candidates were then examined using PCR, RNAi, and in situ hybridization. Primers used to amplify fragments of each gene are given in Supplementary File S1.

Subsequently, we performed BLAST while including the beetle *Tribolium castaneum* and the bee *Apis melifera* and collected sequences from these four species that corresponded to the original reciprocal BLAST hits and, if present, the next most closely related *D. melanogaster* gene. These sets of sequences were aligned using Clustal Omega [76]. The resulting alignments were used for phylogenetic analysis with RAxML and support was estimated with 100 bootstrap replicates [77]. Command line used form: raxmlHPC-PTHREADS -T 4 -f a -x 127,745 -p 453,125 -# 100 -m PROTCATDAYHOFF -s ClustalAlignmentInputGeneX -n OutputGeneX.

Trees were edited using FigTree (v1.4.4) (http://tree.bio.ed.ac.uk/software/figtree/ (accessed on 2 December 2021), and are presented in Supplementary File S2. Some of these analyses identified additional potential orthologs, as the phylogenetic clustering did not necessarily agree with reciprocal BLAST results. These additional candidates will be the subject of future analyses. Sequences that had only one BLAST result per species (Windbeutel, Myd88, Tube, and Bcl3) were not subjected to phylogenetic analysis.

### 2.2. In Situ Hybridization

Ovarioles and embryos were collected, dissected, and processed from wildtype, AsymCX, wasps using standard protocols [8,43].

Probe production and in situ hybridization were performed using standard protocols on ovarioles and embryos in order to characterize normal expression patterns of each transcript of interest during oogenesis and embryogenesis [8,9]. Samples were imaged at 20× magnification on a widefield, compound epi-fluorescent microscope (AXIO IMAGER M2, Zeiss, Jena, Germany).

### 2.3. Parental RNA Interference (pRNAi)

Yellow AsymCx pupae were injected with dsRNA (~1μg/mL in water) designed against each of the transcripts of interest (e.g., *Nv-tollA*, *Nv-dl1*, *Nv-dl2*, *Nv-dl3*, *Nv-dl4, Nv-spz1*) using standard protocols [78]. Injected pupae were allowed to eclose. Adult RNAi wasps were provided honey water for one day post eclosure and allowed to lay eggs. Wasps were reared individually, in isolated, solitary egg laying chambers [78] or socially in large communal egg laying chambers [37]. Fresh hosts were provided daily. Laid eggs were screened (see below) for quantity (clutch size) and viability (embryonic lethality or malformations).

### 2.4. Qualitative Polymerase Chain Reaction (qPCR)

RNA was isolated from 0–4 h (28 °C) knockdown embryos using standard TRIzol-based protocols (Ambion 15596018) and converted into cDNA using the Protoscript First Strand cDNA synthesis kit (NEB 63001), controlling for total RNA input. For each condition, multiple batches of wasps were injected. Embryos were pooled together from multiple egg lays within an injection batch, but not between different batches (biological replicates). Additionally, two cDNA technical replicates were synthesized per biological replicate. Embryos from mock, water-injected embryos were collected and prepared in a similar manner.

To assess knockdown, qPCR was performed on both knockdown and mock-treated embryos. Standard PCR reactions were assembled using the PowerUp SYBR Green Master Mix (Applied Biosystems: A25742). For each sample, reactions were performed using primers specific to the transcript of interest (*Nv-dl1*, *Nv-dl2*, *Nv-dl3*, *Nv-dl4*) and primers specific to a housekeeping gene (*Nv-rp49*). Reactions were performed in triplicate using standard parameters: (50 °C for 2′, 95 °C for 2′, 40 cycles of (95 °C for 15 s, 60 °C for 60 s, plate read, 72 °C for 60 s, plate read), 95 °C for 2′, gradient 60 °C→95 °C (0.2 °C for 1 s). Primer sequences are provided in Supplementary File S1.

Technical replicates (triplicates from both cDNA replicates) for each sample and primer set combination were combined to calculate an average C_T_ value. Delta-Delta C_T_ values were calculated for each knockdown condition and expressed as a relative expression (percentage of wildtype expression) after normalizing to *rp49* levels.

### 2.5. Embryonic Lethality Screening

Overnight egg lays from RNAi wasps were collected and plated onto 1% PBS agar plates. Clutch sizes, the number of embryos laid by an individual wasp in a 24 h period, were recorded. Embryos were then screened for embryonic lethality and developmental malformations after being allowed to develop for 24 h at 28 °C (as described in [79]). Mock, water-injected embryos were also collected, plated, and screened as a control.

## 3. Results

### 3.1. Lack of Expression of Eggshell-Modifying Component Homologs

**Figure 2:**
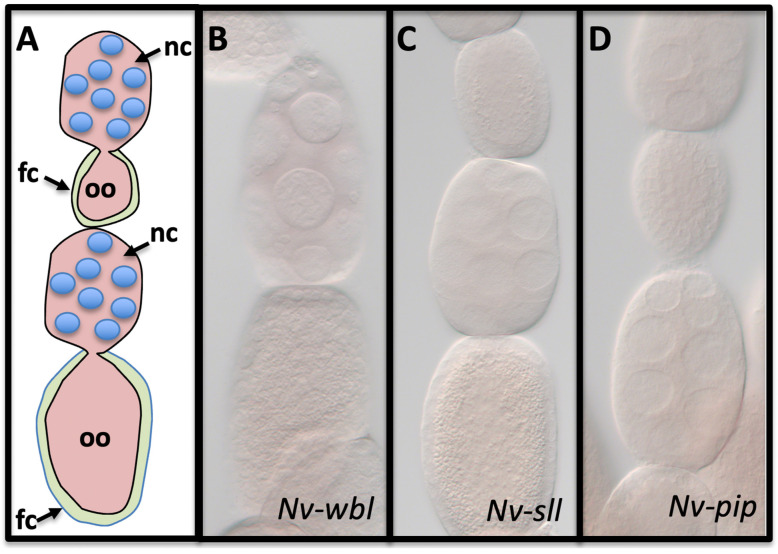
Expression of vitelline membrane altering components. (**A**) Schematic drawing of two *N. vitripennis* egg chambers. fc = follicle cell layer, oo = oocyte, nc = nurse cells. Somatic tissue is shown in green; pink tissue is germline. The upper egg chamber represents early oogenesis, where the oocyte is smaller than the nurse cell compartment, while the lower egg chamber represents late oogenesis. Mid-oogenesis is when the two compartments are roughly the same size. None of *Nv-wbl* (**B**), *Nv-sll* (**C**), or *Nv-pip* (**D**) are expressed detectably in the ovary of *N. vitrpennis*. Mid- and late-stage egg chamber shown.

Since the components and function of the EGF pathway in establishing polarity of the *N. vitripennis* DV axis has already been described, we started our analysis with the homologs of the genes downstream of EGF that transmit the signal from the follicle cells to the protease cascade through sulfation of vitelline membrane components [51]. Single orthologs were found for the crucial factors Wbl, Sll, and Pip using reciprocal BLAST and phylogenetic analysis for Sll and Pip (Supplementary Figures S1 and S2; only a single potential Wbl homolog was found). None of these had detectable expression patterns or levels in *N. vitripennis* ovaries (Figure 2 [80]), in contrast to *D. melanogaster*, where *pip* is expressed strongly in ventral follicle cells and *wbl* and *sll* are expressed throughout the epithelium. Similar to the fly, all three factors lacked detectable expression in the wasp embryo (data not shown). The absence of detectable expression of all three of these specialized components is consistent with our observation that *Nv-pipe* knockdown does not affect DV patterning (not shown). This is also consistent with the lack of *pipe* homolog expression in the follicle cells of the honeybee [81]. Since Pipe has been shown to function in hemimetabolous insect DV patterning [80], it appears that a role for Pipe in DV patterning may have been lost early in hymenopteran evolution.

### 3.2. Novel Paralogs and Expression Patterns of Protease Cascade Components

The components within the perivitelline space are important for activating the Toll signaling pathway on the ventral half of the embryo. Signals from the follicle cells activate a cascade of sequential protein cleavages, leading to the cleavage and activation of the Toll ligand Spätzle. Homologous proteins for all six components (*nudel*, *gastrulation* *defective*, *snake*, *easter, serpin 27A, spätzle*) were identified in *N. vitripennis.* In situ hybridization compared the expression of these homologs in the ovarioles and embryos of *D. melanogaster* and *N. vitripennis*.

### 3.3. Nudel

**Figure 3:**
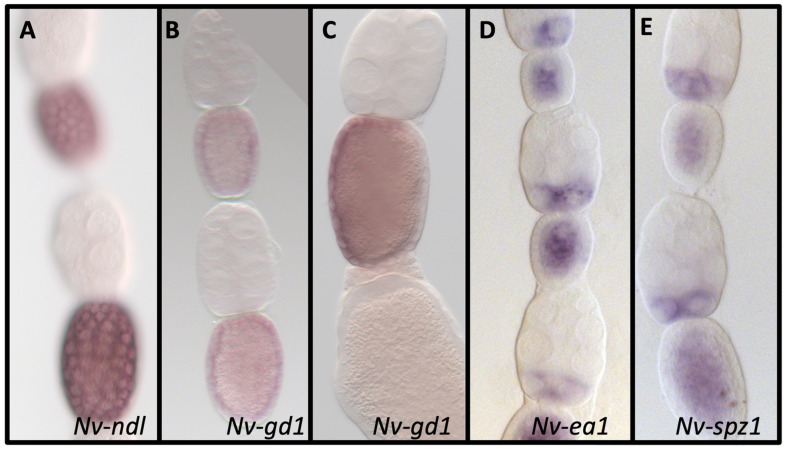
mRNA expression of proteolytic cascade components in the *N. vitrpennis* ovarioles. (**A**) *Nv-ndl* is ubiquitously expressed in follicle cells of mid- and late- (not shown) stage ovarioles, but not in germline cells. (**B**) In early- and mid-stage egg chambers, *Nv-gd1* is expressed throughout the follicle cells. (**C**) Later, *Nv-gd1* becomes restricted to only half of the follicle cells surrounding the late oocyte. (**D**) *Nv-ea1* is expressed broadly in the oocyte from early stages (shown) on, but is restricted to only the posterior nurse cells. (**E**) *Nv-spz1* is ubiquitously expressed in the oocyte and in the nurse cells. Anterior is at the top.

Nudel (Ndl) is exclusively expressed in *D. melanogaster* follicle cells in late oogenesis in the ovary. Initially, we found a single ortholog of Ndl in the *N. vitripennis* genome, based on reciprocal BLAST results. However, a more recent search found an additional sequence (XP_031786376) that also BLASTs back to fly Nudel and clusters with it in phylogenetic analysis (Figure S3). This additional potential paralog will be investigated in future analyses. We termed the originally discovered ortholog (XP_003424379) *Nv-ndl.* Like its *D. melanogaster* ortholog [53,54], *Nv-ndl* is expressed in the follicle cells surrounding the oocyte in late oogenesis, but not in the nurse cells or in the embryo (Figure 3A), consistent with a previous report and the conserved expression of Nudel throughout insects [80].

### 3.4. Gastrulation Defective

**Figure 4:**
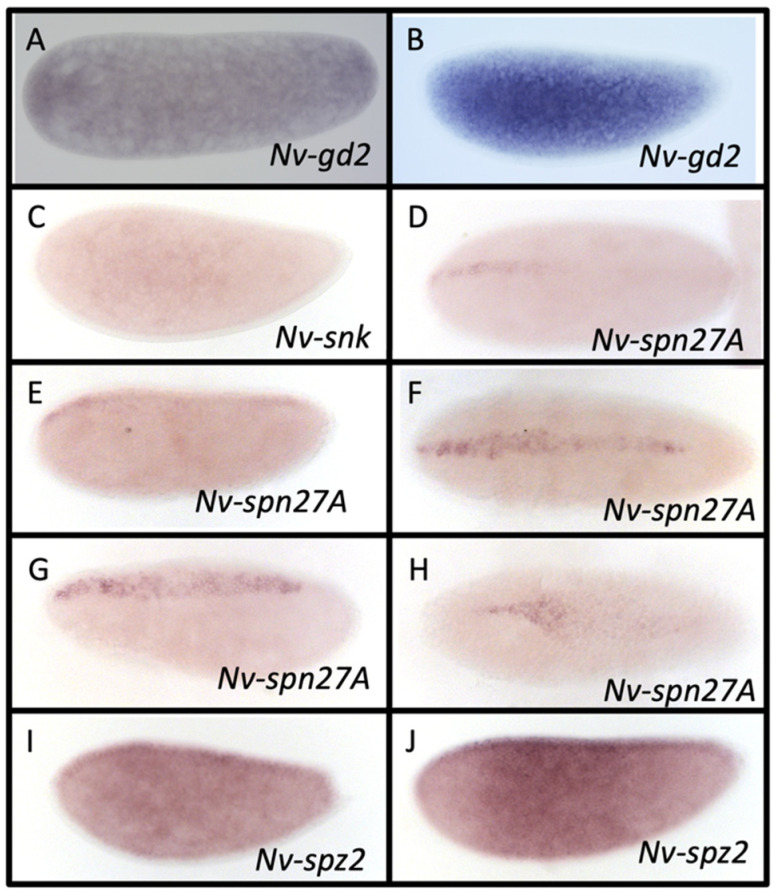
mRNA expression of proteolytic cascade components during *N. vitrpennis* embryogenesis. *Nv-gd2* is initially expressed ubiquitously at low levels in the pre-blastoderm stages (**A**) before being restricted from the cortex and having elevated expression in the cytoplasm/yolk of the syncytial blastoderm (**B**). (**C**) *Nv-snk* is expressed ubiquitously at low levels in the early (pre- to early blastoderm (shown)) embryo. (**D**–**H**) *Nv-spn27A* is initially expressed in a narrow, anterior stripe along the dorsal midline (**D**) before elongating to the posterior pole during the late syncytial blastoderm stage (**E**). The stripe later retracts from the posterior pole (**F**), expands laterally (**G**), and finally becomes localized to the presumptive extraembryonic material after gastrulation (**H**). (**I**,**J**) *Nv-spz2* is ubiquitously expressed throughout the early blastoderm embryo, with elevated expression along the dorsal midline (**I**). Elevated expression then expands laterally, creating a broad ring around the entire circumference of the embryo, from the thoracic to the anterior abdominal segments at the late blastoderm stages (**J**). Anterior is to the left and dorsal is at the top (except in **D**,**F**,**H**; dorsal views).

Gastrulation defective (Gd) is the most upstream component of the cytoplasmic protease cascade that leads to cleavage of Spz. *Dm-gd* is expressed in the oocyte, the surrounding follicle cells, and later on in the embryo [55]. Two paralogs of *Dm-gd* were found in *N. vitripennis* based on reciprocal BLAST results and phylogenetic analysis (Supplementary Figure S4, which also suggests that CG9649 is paralogous to *gd* in the fly)*. Nv-gd1* is expressed throughout the oocyte and follicle cells early on (Figure 3B). In mid and late egg chambers, mRNA expression is restricted to a single side of the follicle epithelium (Figure 3C). *Nv-gd1* is not expressed in the embryo. *Nv-gd2* is neither expressed in the oocyte nor the follicle cells (not shown), but is expressed ubiquitously at low levels in the early embryo and then at higher levels in the yolk of the syncytial blastoderm (Figure 4A,B). Thus, together the two *N. vitripennis* paralogs recapitulate the full expression of *D. melanogaster gd*.

### 3.5. Snake

*D. melanogaster snk* is expressed in the oocyte, and its protein is secreted into the perivitelline space and acts upstream of Easter [21]. Reciprocal BLAST results supported the protein XP_008207491.1 as the most likely *N. vitripennis* ortholog of Snake (hereafter referred to as *Nv-snk*). However, Nv-Snk is more similar to the *N. vitripennis* protein XP_031786740.1 than it is to any *D. melanogaster* sequence, suggesting that the two genes in *N. vitripennis* split from an ancestral *Nv-snk*. Contrary to this, XP_031786740.1 is a reciprocal best BLAST hit with the relatively unknown *D. melanogaster* proteins encoded by the genes CG11841 and CG11842, indicating a set of two ancestral *snk*-like lineages, and we provisionally refer to the gene encoding XP_031786740.1 as *Nv-snk2*. *Nv-snk* and *Nv-snk2* are barely detectable in the ovaries (not shown) and only *Nv-snk* shows weak expression in the early embryo (Figure 4C). Phylogenetic analysis of genes with similarity to Snake suggests several other candidates that might be part of the Snake lineage and which could play a role in the *N. vitripennis* system (Supplementary Figure S5).

### 3.6. Easter

Easter (Ea) is the protease that cleaves Spätzle, producing the active Toll ligand [82]. Melanization protease 1 (MP1) and the Spätzle processing enzyme (Spe) are the top BLAST hits of Ea, and are likely paralogs in *D. melanogaster* (Supplementary Figure S6), but only Ea has been shown to act in the DV patterning pathway [21]. We found two proteins that are reciprocal best hits to Ea, MP1, and Spe in the *N. vitripennis* genome. We named them *Nv-ea1* and *Nv-ea2,* and they are examined here. Phylogenetic analysis revealed a very complex set of relationships, and additional potential paralogs of *easter* may be present (Supplementary Figure S6). *Nv-ea1* is expressed strongly throughout the oocyte and the posterior region of the nurse cells (Figure 3D) and is only weakly detected in the embryo (not shown). *Nv-ea2* was at very low or undetectable levels in both the ovary and embryo (not shown).

### 3.7. Serpin27A

Serpin27A (Spn27A) is an important regulator of the protease cascade and controls its activity over time [21]. A single ortholog was found in *N. vitripennis (Nv-spn27A)* via BLAST and phylogenetic analysis (Supplementary Figure S7). *Nv-spn27A* is not expressed in the ovarioles (not shown), but is expressed in a dynamic pattern in the embryo (Figure 4D–H). mRNA is first localized in a short, narrow stripe at the dorsal midline, near the anterior pole of the embryo (Figure 4D). This expression domain then expands to the posterior pole, retracts back from the posterior pole, and then widens laterally (Figure 4E–G). This lateral expansion increases during gastrulation until it is expressed in the entire presumptive serosa (Figure 4H). This expression pattern is quite different from what is seen in the fly. *Dm-spn27A* is expressed in the oocyte and the early embryo. However, expression remains constant and ubiquitous through the cellular blastoderm stage.

### 3.8. Spätzle

Spätzle is the target of the protease cascade and sets up the gradient of Toll pathway activation and is secreted from the oocyte [83]. We identified two potential paralogs of Spz in the *N. vitripennis* genome with reciprocal BLAST and support from phylogenetic analysis (Supplementary Figure S8), indicating a duplication in the wasp lineage. *Nv-spz1* is expressed in the ovarioles in a similar pattern to that of *Nv-ea1*, throughout the oocyte and localized to the posterior of the nurse cells (Figure 3E). Expression is also similarly lacking in the follicle cells and the embryo (not shown). *Nv-spz2* is not expressed in any portion of the ovarioles (not shown), but is expressed in the embryo. Expression is ubiquitous throughout the embryo, but elevated along the dorsal midline (Figure 4I). Elevated expression then expands to a broad lateral domain encircling the entire trunk of the embryo in later blastoderm stages (Figure 4J). This late and dynamic expression seen in *Nv-spz2* has not been described in *D. melanogaster*.

### 3.9. Toll Receptors

In *D. melanogaster,* the Toll receptor receives positional information from the follicle cells and perivitelline space through its ligand Spätzle. Toll is then responsible for activating the signaling cascade that will fully polarize the embryo.

**Figure 5:**
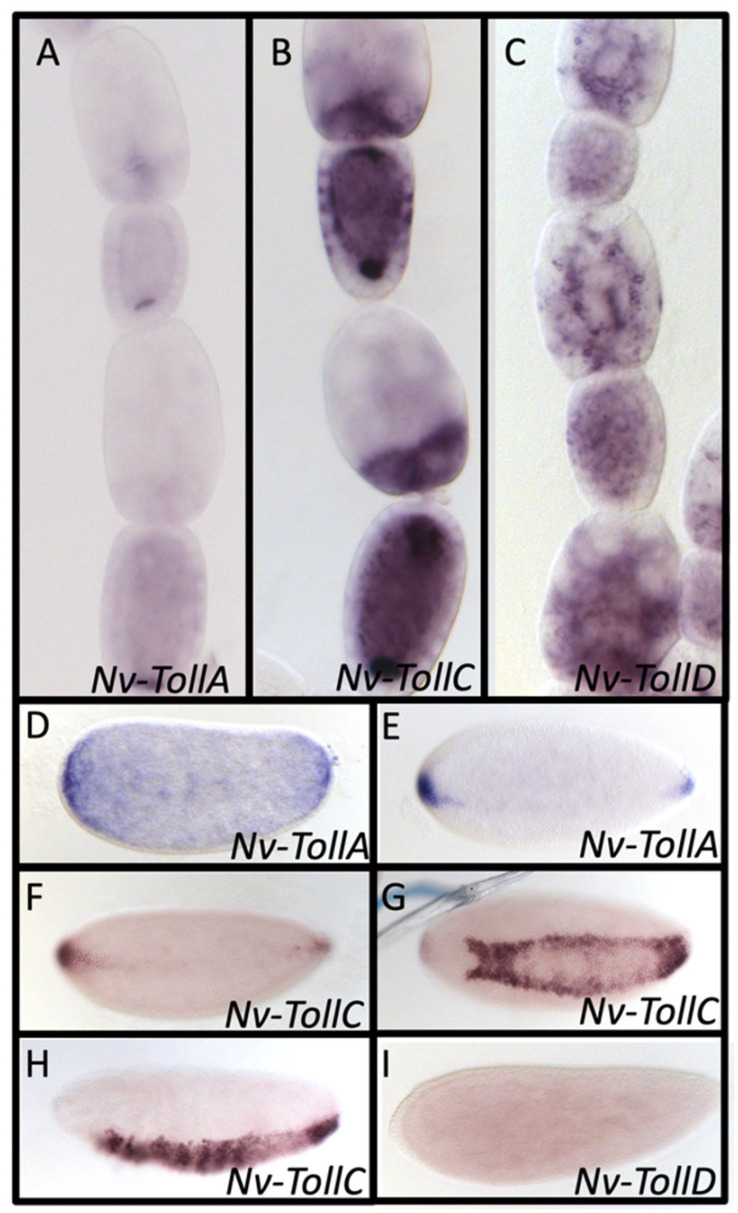
mRNA expression of Toll paralogs in *N. vitrpennis* ovarioles and embryos. (**A**–**D**) Egg chambers at mid to late oogenesis shown. (**A**) *Nv-TollA* is localized posteriorly in the oocyte. (**B**) *Nv-TollC* is strongly enriched at both the anterior and posterior poles of the oocyte and has elevated expression in the posterior of the nurse cells. (**C**) *Nv-TollD* is ubiquitously expressed in the oocyte and nurse cells. (**D**,**E**) *Nv-TollA* is initially ubiquitously expressed, with elevated expression at the anterior and posterior ends in the very early blastoderm stage (**D**). Expression then becomes restricted to just the pole, before expanding along the ventral midline in mid to late blastoderm stages (**E**). (**F**–**H**) *Nv-TollC* is initially expressed at the anterior and posterior poles at mid blastoderm before elongating along the ventral midline in the late blastoderm (**F**). The stripe later outlines the mesoderm–ectoderm border (**G**). Expression expands ventrally filling in the presumptive mesoderm at gastrulation (**H**). (**l**) *Nv-TollD* is expressed ubiquitously at low levels (mid blastoderm stage shown).

There is a single ortholog of the Toll receptor responsible for activating Dorsal in embryonic patterning and immunity. In contrast, we found four Toll paralogs in the *N. vitripennis* genome that reciprocally BLAST to fly Toll and which are direct orthologs of *Dm-Toll*, based on phylogenetic analysis (Supplementary Figure S9), distinct from the larger family of Toll-like receptors found among insects [84,85,86]). Three of these (*Nv-TollA*, *C*, *D*) are expressed in both the oocyte and embryo; however, the details of their expression vary. *Nv-TollA* is expressed in the posterior nurse cells and throughout the oocyte, with strong posterior enrichment and localization (Figure 5A). *Nv-TollC* is expressed more strongly in the nurse cells and highly enriched in the most posterior ones, and the mRNA is highly enriched at both the anterior and posterior poles of the oocyte (Figure 5B). *Nv-TollD* is more ubiquitously expressed throughout the nurse cells and accumulates throughout the oocyte (as opposed to the localization at the poles in *Nv-TollA* and *C*) (Figure 5C).

In the early blastoderm embryo, *Nv-TollA* is ubiquitously expressed at low levels, with higher levels of expression in both the anterior and posterior polar regions (Figure 5D). Later, expression becomes tightly restricted to the two poles and then starts to form a very thin stripe along the ventral midline (Figure 5E). *Nv-TollC* is initially expressed at the anterior and posterior poles of the early *N. vitripennis* blastoderm and also shows a narrow ventral midline stripe early on (Figure 5F), similar to *Nv-TollA*. This stripe quickly evolves to a domain that outlines the borders of the presumptive mesoderm (Figure 5G). Once gastrulation begins, expression is strong throughout the entire presumptive mesoderm (Figure 5H). *Nv-TollD* is expressed ubiquitously at low levels in the embryo (Figure 5I). *Nv-TollB* is neither expressed during oogenesis nor embryogenesis.

This asymmetric localization of *Nv-Toll* mRNAs in both the ovary and embryo is unexpected. *Dm-Toll* is also maternally expressed in the oocyte and embryo, but it is ubiquitously expressed through the blastoderm stages similar to the expression of *NvTollD* [87,88,89,90]. It is not until gastrulation that zygotic *Toll* starts to be differentially expressed [87,88,89,90].

### 3.10. Membrane-Interacting Mediators of Toll Signaling

**Figure 6:**
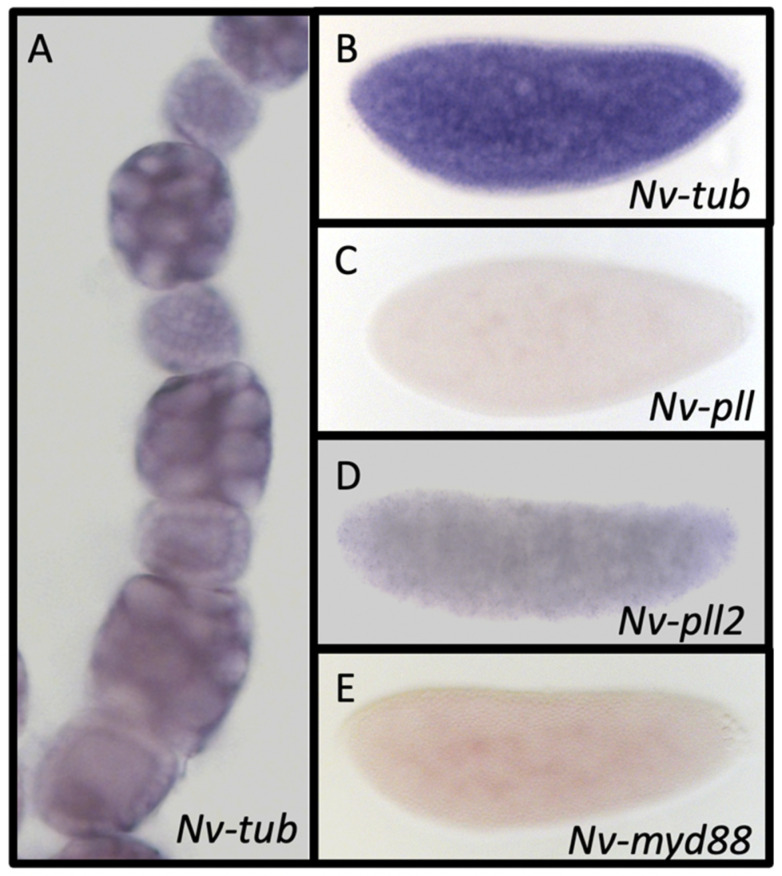
Membrane-associated components’ expression in ovaries and embryos. (**A**) *Nv-tub* is expressed in nurse cells, oocytes, and follicle cells from early stages of oogenesis onward (early stage shown). (**B**–**D**) Membrane-associated components (**B**) *Nv-tub*, (**C**) *Nv-pll*, (**D**) *Nv-pll2*, and (**E**) *Nv-myd88* are ubiquitously expressed throughout early embryogenesis (mid blastoderm stages shown).

Several components interact with the membrane-bound activated Toll receptor and transmit the signal into the cell. *D. melanogaster myeloid differentiation primary response gene 88* (*Myd88*) is required to localize Tube to the membrane upon Toll activation. In turn, Tube binds to Pelle, which leads to Pelle phosphorylation. Phosphorylation of Pelle leads to phosphorylation of Cactus, and the translocation of Dorsal to the nucleus [11,71]. Single orthologs were detected in *N. vitripennis* for Tube and Myd88 with reciprocal BLAST, and two paralogs were confirmed for *N. vitripennis* Pelle (Nv-Pll) (Supplementary Figure S10). *N. vitripennis tube* (*Nv-tub*) is expressed in both ovaries and the early embryo (Figure 6A,B). The two *Nv-pll* paralogs, as well as *Nv-Myd88*, are not detected by in situ hybridization in the ovary (not shown), but are expressed at low levels in the early embryo (Figure 6C–E).

### 3.11. Dorsal Inhibitors

In *D. melanogaster*, Cactus binding prevents Dorsal from translocating to the nucleus. Toll signaling leads to Cactus phosphorylation and degradation, freeing Dorsal. While a single *cactus* gene performs this function in the fly, three genes orthologous to Cactus (*Nv-cact1, 2, and 3*) were found in the *N. vitripennis* genome by reciprocal BLAST and phylogenetic analysis (Supplementary Figure S11).

**Figure 7:**
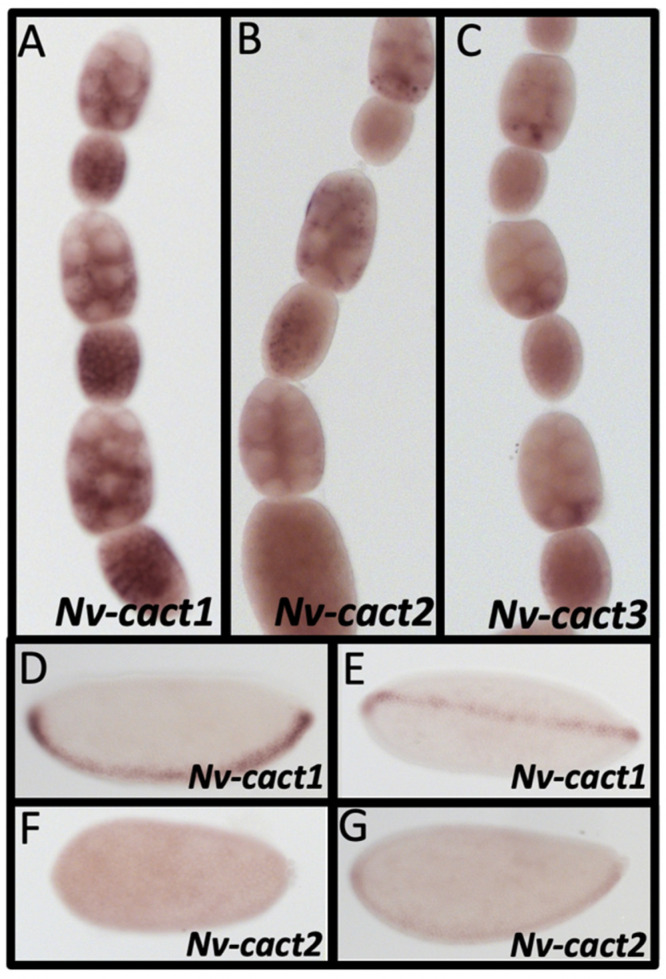
Expression of *Nv-cact* paralogs. (**A**) *Nv-cact1* is expressed in the oocyte, follicle cells, and most of the nurse cell compartment throughout early, mid, and late oogenesis (early and mid stages shown). (**B**) *Nv-cact2* is ubiquitously expressed in the oocyte and nurse cells throughout oogenesis (mid and late stages shown). (**C**) *Nv-cact3* is ubiquitously expressed in the oocyte and nurse cells, with enhanced expression in posterior nurse cells (mid oogenesis stages shown). (**D**,**E**) In early blastoderm embryos, *Nv-cact1* is expressed in a narrow stripe along the ventral midline (**D**) which remains throughout the late blastoderm stage (**E**). (**F**,**G)** *Nv-cact2* is initially expressed ubiquitously in the early blastoderm (**F**), before gaining elevated expression along the ventral midline in late oogenesis (**G**).

All three *Nv-cact* paralogs are expressed in the nurse cells and oocyte, with slightly different patterns in the nurse cells for each (Figure 7A–C). *Nv-cact1* is also expressed in the follicle cells (Figure 7A). In addition to the ubiquitous maternal contribution, *Nv-cact1 and Nv-cact2* have patterned zygotic expression in the embryo. *Nv-cact1* is strongly expressed along the ventral midline (Figure 7D). This narrow stripe persists from the early blastoderm until the onset of gastrulation (Figure 7E). *Nv-cact2* is ubiquitously expressed in the early embryo (Figure 7F). Later blastoderm stages have slightly elevated expression along the ventral midline, similar to, but slightly weaker than, what is seen in *Nv-cact1* (Figure 7G). These patterns may indicate feedback control of *Nv-cact1* and *2* by the Toll signaling pathway, as seen in other insects [91,92]. *Nv-cact3* is not expressed in the embryo.

**Figure 8:**
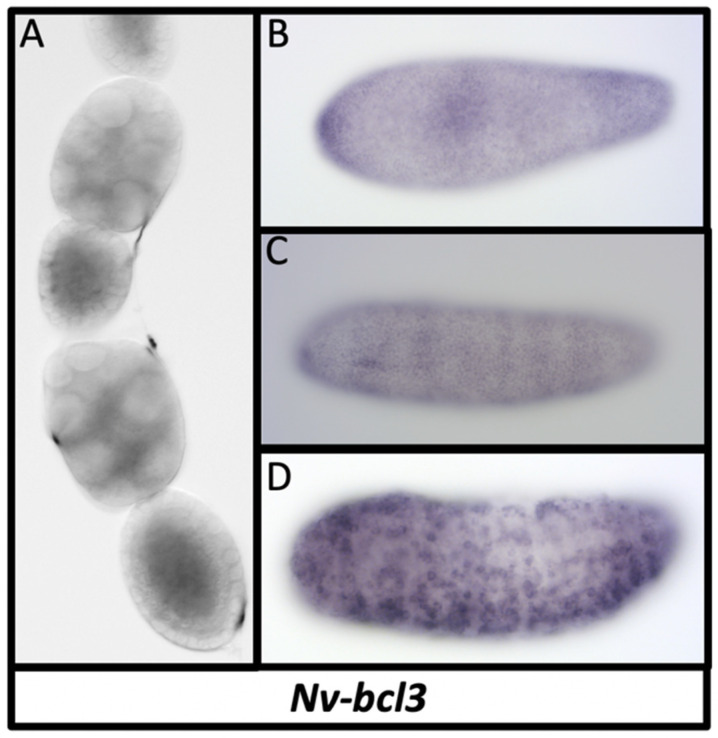
Expression of *Nv-bcl3* in ovaries and embryos. (**A**) *Nv-bcl3* is ubiquitously expressed in oocytes, lacking in the follicle cells, and is restricted to the posterior of the nurse cells (early to mid oogenesis shown). (**B**) In the early blastoderm, *Nv-bcl3* is expressed ubiquitously with variation in intensity along the AP axis. (**C**) Expression evolves into broad stripes with fuzzy borders into late blastoderm stages. (**D**) After gastrulation, *Nv-bcl3* is expressed in scattered cells of unknown fate.

We also found an additional potential Dorsal inhibiting factor in the *N. vitripennis* genome. It appears to be most closely related to the B-cell lymphoma-3 (Bcl3) genes in vertebrates, which are distantly related to the IκB factors, like Cactus, and perform similar roles in controlling NfκB nuclear translocation [93]. We also found homologs of Bcl3 in other insects, including the bee *Apis meliferra* and the beetle *Tribolium castaneum*. *Nv-bcl3* is expressed in the nurse cells, strongly but ubiquitously in the oocyte, but not in the follicle cells (Figure 8A). In the embryo, *Nv-bcl3* is expressed dynamically in a weakly periodic fashion along the AP axis, with broad, alternating regions of high and low expression that become more stripe-like over time (Figure 8B,C). After gastrulation, *Nv-bcl3* is expressed in scattered cells across the ectoderm (Figure 8D).

### 3.12. Duplications in Dorsal

Dorsal nuclear translocation is the functional output of Toll signaling. In the fly, a single *dorsal* gene, expressed maternally, performs the patterning function. In *N. vitripennis,* we found four paralogous genes related to *dorsal* (*Nv-dl1-4*) (Supplementary Figure S12).

**Figure 9:**
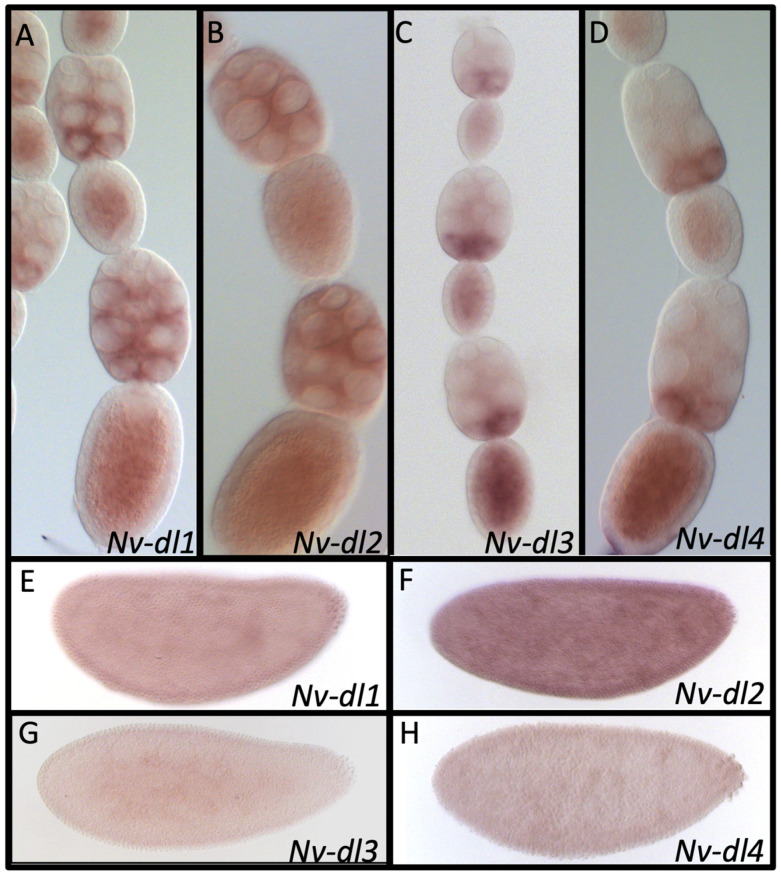
mRNA expression of *dorsal* paralogs in *N. vitrpennis* ovarioles and embryos. (**A**) *Nv-dl1* is expressed ubiquitously in the oocyte and most of the nurse cells, except for the most anterior ones (mid to late oogenesis shown) (**B**) *Nv-dl2* is ubiquitously expressed in the oocyte and nurse cells. (**C**) *Nv-dl3* is ubiquitously expressed in the oocyte, and is strongly upregulated in the posterior of the nurse cells (early to mid stages shown). (**D**) *Nv-dl4* is expressed in the oocyte and the nurse cells, with expression concentrated in posterior nurse cells (mid to late stages shown). *Nv-dl1* (**E**), *Nv-dl2* (**F**), *Nv-dl3* (**G**), and *Nv-dl4* (**H**) are ubiquitously expressed in the embryo (early blastoderm stages shown). Anterior is at the top for ovaries and to the left for embryos.

All four *N. vitripennis dorsal* transcripts are expressed maternally in the germline cells (Figure 9A–D). *Nv-dl1* shows uniform expression in the nurse cells and oocyte (Figure 9A), while *Nv-dl2* appears to be present in a posterior-to-anterior gradient in the nurse cells (Figure 9B). *Nv-dl3* and *Nv-dl4* show strong concentration in the posterior nurse cells adjacent to the oocyte (Figure 9C,D). These differences indicate distinct modes of regulation of these paralogs in the germline, the functional significance of which is at present unclear. All four paralogs are expressed uniformly at the blastoderm stage (with very low levels seen in Nv-dl3) and show no patterned expression (Figure 9E–H).

### 3.13. Functional Analysis of Pathway Components

#### Functional Analysis of Nv-Dorsal Paralogs

While four *Nv*-Toll paralogs were found, it has already been shown that the knockdown of *Nv*-*TollA* by pRNAi results in the dorsalization of the embryo and high levels of embryonic lethality [36]. However, knocking down *Nv*-*TollB*, *C*, or *D* in a similar way did not result in these or any other observable developmental phenotype.

**Figure 10:**
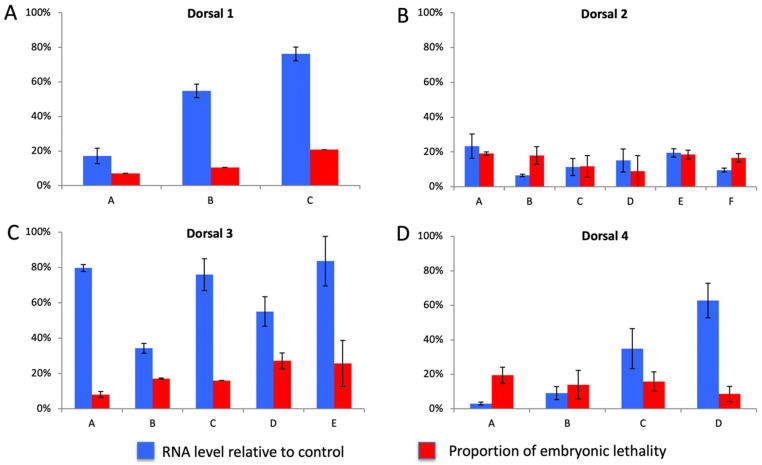
mRNA expression and embryonic lethality following pRNAi. (**A**–**D**) Relative transcript expression (blue) following pRNAi as a percentage of wildtype expression compared to the percentage of eggs laid that failed to develop into larva (embryonic lethality, red) from the same batch of wasps following pRNAi treatment. The *X*-axis represents different batches of RNAi injected wasps. Wasps were injected with dsRNA targeting *dorsal1* (**A**), *dorsal2* (**B**), *dorsal3* (**C**), *dorsal4* (**D**). Error bars represent standard error.

In order to similarly determine which of the four *Nv*-Dorsal paralogs is functionally equivalent to *Dm*-Dl, each paralog was also knocked down by pRNAi and screened for phenotypes. In *D. melanogaster*, Toll and Dorsal mutants both cause a similar dorsalizing phenotype, therefore it is predicted that the functional *N. vitripennis* paralog would cause similar phenotypes, as seen in the *Nv-Toll* knockdowns [21]. Following pRNAi, none of the predicted phenotypes were observed for any of the four paralogs. The average embryonic lethality observed for each paralog was under 15% and in situ hybridization revealed no clear disruption of DV patterning (not shown). Quantitative PCR confirmed that the lack of observed phenotypes was not due to a failure in knockdown, as transcript levels were reduced to below 30% of wildtype levels in multiple clutches and for each paralog (Figure 10). While both degree of knockdown and frequency of lethality were variable, there was no clear correlation between the two (i.e., strong knockdowns did not show higher embryonic lethality, and weaker knockdowns still showed lethality well above control levels). These results cannot differentiate between incomplete knockdown and redundant function among the paralogs as the explanation for the lack of significant phenotypes when knocking down *Nv-dl* paralogs. Stronger loss of function disruptions (e.g., CRISPR-induced frameshifts) and multi-target approaches will be necessary to determine which Dorsal paralogs function in *N. vitripennis* embryonic patterning.

### 3.14. Functional Analysis of Other DV Patterning Components

Previous work has demonstrated that the reduction of EGF signaling by pRNAi leads to a ventralized, partially duplicated axis phenotype in *N. vitrpennis* embryos [43]. In *D. melanogaster* a similar, but stronger, effect is mediated by expansion of the *pipe* expression domain [45], allowing unrestrained cleavage of *spz* [94]. Given the lack of detectable, patterned expression of *Nv-pipe*, it seemed unlikely that *Nv-pipe* would play a role in mediating the EGF signal. Indeed, neither *Nv-pipe* nor *Nv-wbl* pRNAi led to patterning defects or reduced viability in embryos, further indicating that this part of the pathway has been deleted in *N. vitrpennis*.

Preliminary analysis of the protease cascade has not resulted in clear results for the functional conservation of this part of the pathway. No phenotypes were recovered in preliminary pRNAis against *Nv-gd1*, *Nv-gd2*, *Nv-snk1*, *Nv-ea1*, or *Nv-ea2* (not shown). We believe that this is the result of the combination of incomplete knockdown and possible redundant functionality of paralogs for all of these proteases. *Nv-ndl* was the exception in giving an embryonic lethal phenotype, but the knockdown resulted in defective eggshells, and no embryonic material could be obtained to determine whether patterning was disrupted.

**Figure 11:**
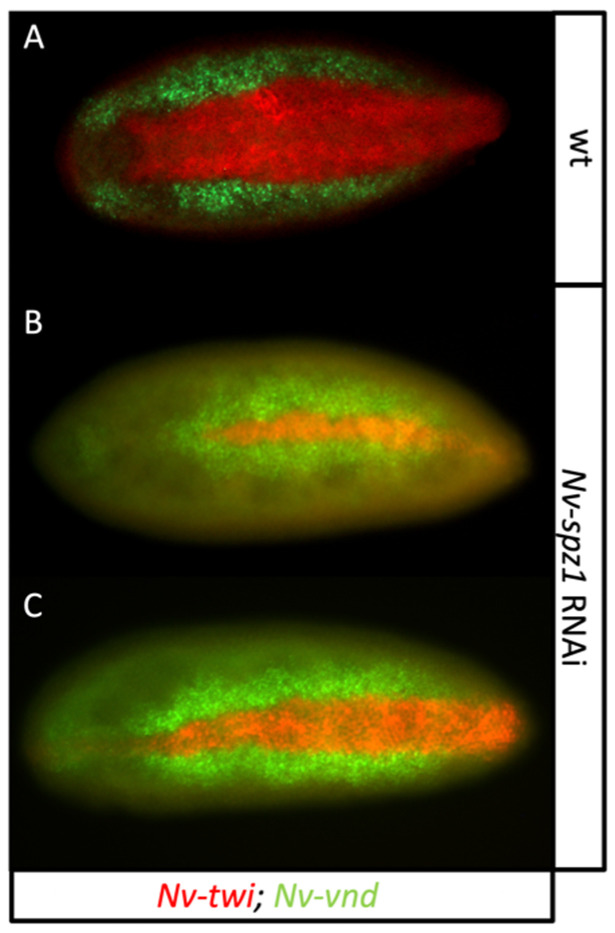
RNAi knockdown of *Nv-spz1.* (**A**) Ventral view of *Nv-twi* and *Nv-vnd* (red and green, respectively) in a wildtype embryo in late blastoderm stage. (**B**,**C**) A strong (**B**) and a weaker (**C**) *Nv-spz1* dorsalizing phenotype in late blastoderm embryos.

Downstream of the protease cascade, at least one of the *Nv-spz* paralogs, *Nv-spz1*, is required for DV patterning, and phenotypes similar to weak *Nv-TollA* knockdowns were obtained with *Nv-spz1* pRNAi (Figure 11). Only the previously published *Nv-TollA* gave dorsalizing phenotypes, indicating that it is the primary receptor mediating *Nv*-Spz1 signaling to the embryo. The potential role of the presumed membrane-associated mediators of *Nv*-Toll signaling, *Nv-myd88*, *-pll1*, *-tub,* were also investigated. Their role in DV patterning could not be assessed, as the injected wasps were all sickly, with short life-spans, and did not lay eggs. This is likely due to the crucial roles of these genes in innate immunity. The knockdown of *Nv-pll2* did not result in sterile wasps. The embryos laid by these wasps did not hatch and had larval deformities commonly seen in other dorsalizing knockdown phenotypes (not shown). Misexpression of downstream DV genes following RNAi still needs to be investigated to confirm this defect in polarity. Finally, pRNAi against the *Nv-cact* paralogs and *Nv-bcl3* resulted in normal embryos, and no DV patterning defects were detected (not shown). Again, we believe this is likely due to incomplete knockdowns and redundancy among paralogs. A summary of these experiments is presented in Table 1.

## 4. Discussion

Here we have shown that orthologs of all known components of the Toll signaling pathway are present and well conserved in the wasp *N. vitrpennis.* For most of the fly Toll pathway genes, at least one *N. vitripennis* ortholog is expressed in a pattern consistent with having a conserved function. Unfortunately, we could not confirm the presence of conserved function for many of the identified components in this preliminary analysis. More focused and intensive functional testing will be required to confidently ascribe or eliminate potential functions for many of these factors.

An exception to the general rule of conserved expression is the cassette of proteins needed for sulfation of the vitelline membrane: Sll, Wbl, and Pipe. While present in the genome, none of these are expressed in the ovary of *N. vitrpennis,* and do not give phenotypes. Based on the recent discovery of a *Drosophila*-like function of Pipe in the cricket [80], it appears that this pathway has been deleted from the DV patterning hierarchy in the *N. vitrpennis* lineage. The absence of *pipe* expression in the follicle cells in the honeybee indicates that the loss likely happened early in hymenopteran evolution [81]. Interestingly, a recent investigation of vitelline membrane proteins in *N. vitrpennis* [95] found that their knockdown leads to phenotypes at the cuticular level quite similar to those where the DV axis is disrupted ([43,96], J.A.L., personal observations). This indicates that vitelline membrane components still play a role in establishing DV polarity, but the mechanism and potentially the effect may be different.

We also observed large scale gene duplication resulting in multiple paralogs of several components of the system, most notably four *Toll* paralogs, four *dorsal* paralogs, and three *cactus* paralogs. The functional relevance of so many paralogs is so far not clear. It is not a general feature of *N. vitrpennis* signaling pathways, as the wasp BMP pathway exhibited very few lineage-specific paralogs [37]. Are these duplication events adding robustness and protection to vital portions of the pathway, potentially through subfunctionalization or specialization of the various paralogs? Or are they paired with modified/diversified expression pattern domains because they are evolving to meet some species-specific function or need? In depth analysis and more robust gene disruption techniques will be needed to answer these questions.

Perhaps the biggest unanswered question is: how is the EGF signal from the oocyte to the follicle cells transmitted to the embryo? We have shown that EGF-mediated asymmetry signals are conserved and required to polarize the embryo. Here we have also shown that Spz1 is likely the ligand primarily responsible for embryonic Toll activation. So far, however, how the EGF polarity information is converted into localized production of active Spz is unknown. We do know that Nv-Pip is not involved. Have novel proteins been coopted into the pathway to fill Nv-Pip’s void? Or is a completely new mechanism in place to relay the EGF signal from the follicle cells to the oocyte? The expression of *Nv-gd1* in a subset of follicle cells is potentially significant, since fly Gd protein becomes enriched at the ventral side of the eggshell in a Pipe-dependent manner, and thus plays a crucial role in polarizing the protease cascade and producing ventral cleavage of Spz [97]. If Nv-Gd1 protein localization reflects its mRNA, this could provide a method to polarize the embryo in the absence of Nv-Pip function. While preliminarily *Nv-gd1* knockdown gave no phenotype, future studies could employ more complete knock-out approaches, such as CRISPR, to test this idea more conclusively.

In general, it is clear that new approaches are needed to rigorously test the function of Toll signaling components in *N. vitripennis*, which will then allow more meaningful comparisons with other insect species with the goal of understanding the evolutionary significance of this ancient pathway in the diversity of insects.

## Figures and Tables

**Table 1:** Summary of Toll Pathway component expression in the fly and wasp. Tissue-based expression domains in both the fly and wasp are provided for each pathway component based on previous [88,89,90] and current studies (color has been added to emphasize similarities/differences). RNAi phenotypes observed in the wasp include: X = no phenotype, D = dorsalized, I = immune/sick wasps, F = flaccid/fragile eggs, N/A = not tested.

*Drosophila* Protein	*Nasonia* Ortholog	*Drosophila* Transcript Expression			*Nasonia* Transcript Expression			RNAi
**Follicular Epithelium Components**		Follicle cells	Oocyte	Early Embryo	Follicle cells	Oocyte	Early Embryo	**Phenotype**
Windbeutel	Nv-Wbl	YES	NO	NO	NO	NO	NO	X
Slalom	Nv-Sll	YES	NO	NO	NO	NO	NO	N/A
Pipe	Nv-Pip	YES	NO	NO	NO	NO	NO	X
**Perivitelline Space Components**								
Nudel	Nv-Ndl	YES	NO	NO	YES	NO	NO	F
Gastrulation defective	Nv-Gd	YES	YES	YES	YES	YES	NO	X
	Nv-Gd2	-	-	-	NO	NO	YES	X
Snake	Nv-Snk	NO	YES	YES	NO	NO	YES	X
	Nv-Snk2	-	-	-	NO	NO	NO	N/A
Easter	Nv-Ea1	NO	YES	YES	NO	YES	NO	X
	Nv-Ea2	-	-	-	NO	NO	NO	X
Serpin27A	Nv-Spn27A	NO	YES	YES	NO	NO	YES	N/A
Spatzle	Nv-Spz1	NO	YES	YES	NO	YES	NO	D
	Nv-Spz2	-	-	-	NO	NO	YES	D
**Toll Receptors**								
Toll	Nv-TollA	NO	YES	YES	NO	YES	YES	D
	Nv-TollB	-	-	-	NO	NO	NO	X
	Nv-TollC	-	-	-	NO	YES	YES	X
	Nv-TollD	-	-	-	NO	YES	YES	I
**Intracellular Components**								
Tube	Nv-Tub	NO	YES	YES	NO	YES	YES	I
Pelle	Nv-Pll1	NO	YES	YES	NO	NO	YES	I
	Nv-Pll2	-	-	-	NO	NO	YES	D
Myd88	Nv-Myd88	NO	YES	YES	NO	NO	YES	I
Cactus	Nv-Cact1	NO	YES	YES	YES	YES	YES	X
	Nv-Cact2	-	-	-	YES	YES	YES	X
	Nv-Cact3	-	-	-	YES	YES	NO	X
B-cell lymphoma 3 (mammalian)	Nv-Bcl3	N/A	N/A	N/A	NO	YES	YES	X
Dorsal	Nv-Dl1	NO	YES	YES	NO	YES	YES	X
	Nv-Dl2	-	-	-	NO	YES	YES	X
	Nv-Dl3	-	-	-	NO	YES	YES	X
	Nv-Dl4	-	-	-	NO	YES	YES	X

## Supplementary Materials

The following supporting information can be downloaded at: https://www.mdpi.com/article/10.3390/jdb10010007/s1, Supplementary File S1: Table S1: Primers used in this study. Supplementary File S2: Figures S1–S12: Phylogenetic trees of candidate genes analyzed in this study.
