# Supplementary material for: Expression and Function of Toll Pathway Components in the Early Development of the Wasp Nasonia vitripennis
Source: J Dev Biol. 2022 Jan 26;10(1):7. doi: 10.3390/jdb10010007 (PMC8883978; doi:10.3390/jdb10010007)
Supplement: Supplementary file 1 [file jdb-10-00007-s001.zip › Supplemental File S2-trees.pdf]

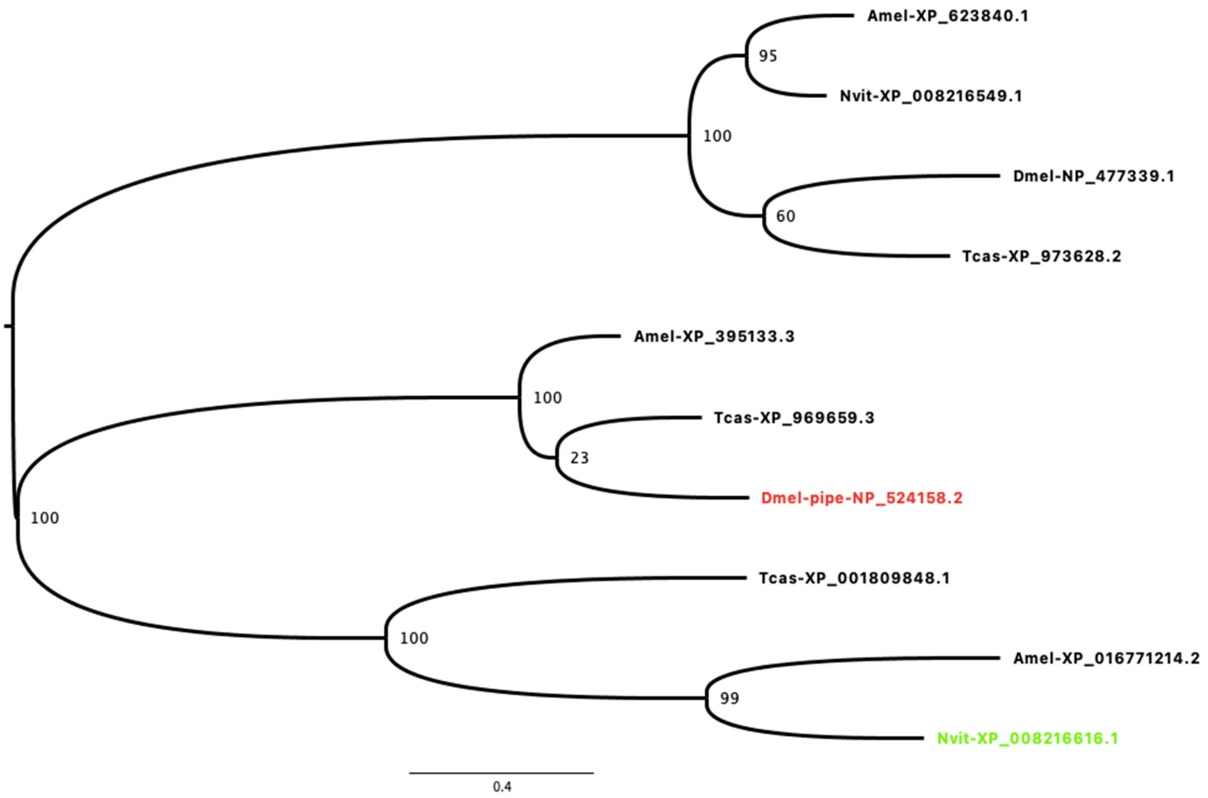

Supplementary Figure S1. Phylogenetic analysis of Pipe. Likely Nasonia ortholog of D. melanogaster Pipe (red) in green. Node labels are bootstrap support.

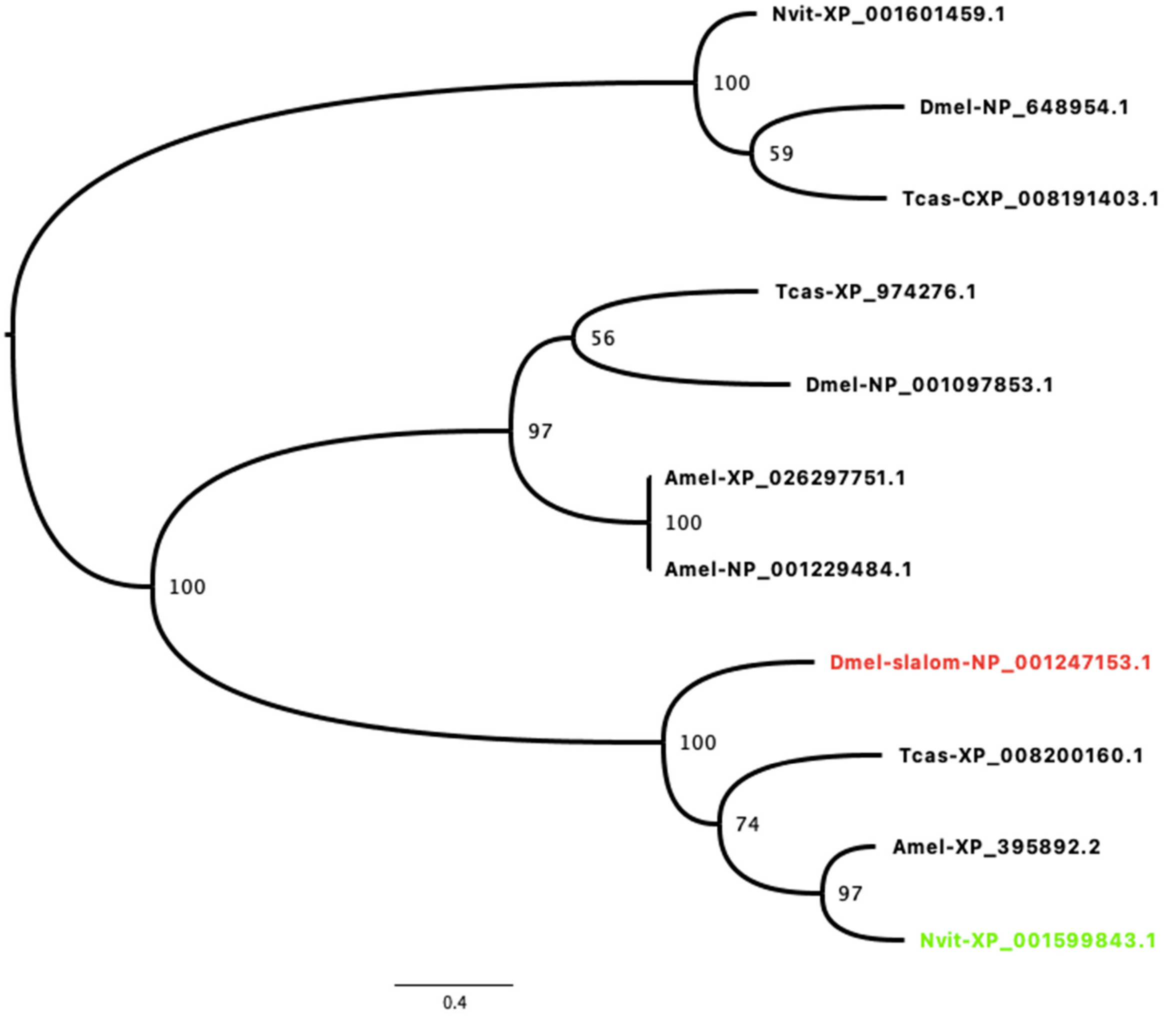

Supplementary Figure S2. Phylogenetic analysis of Slalom. Likely Nasonia ortholog of D. melanogaster Slalom (red) in green. Node labels are bootstrap support.

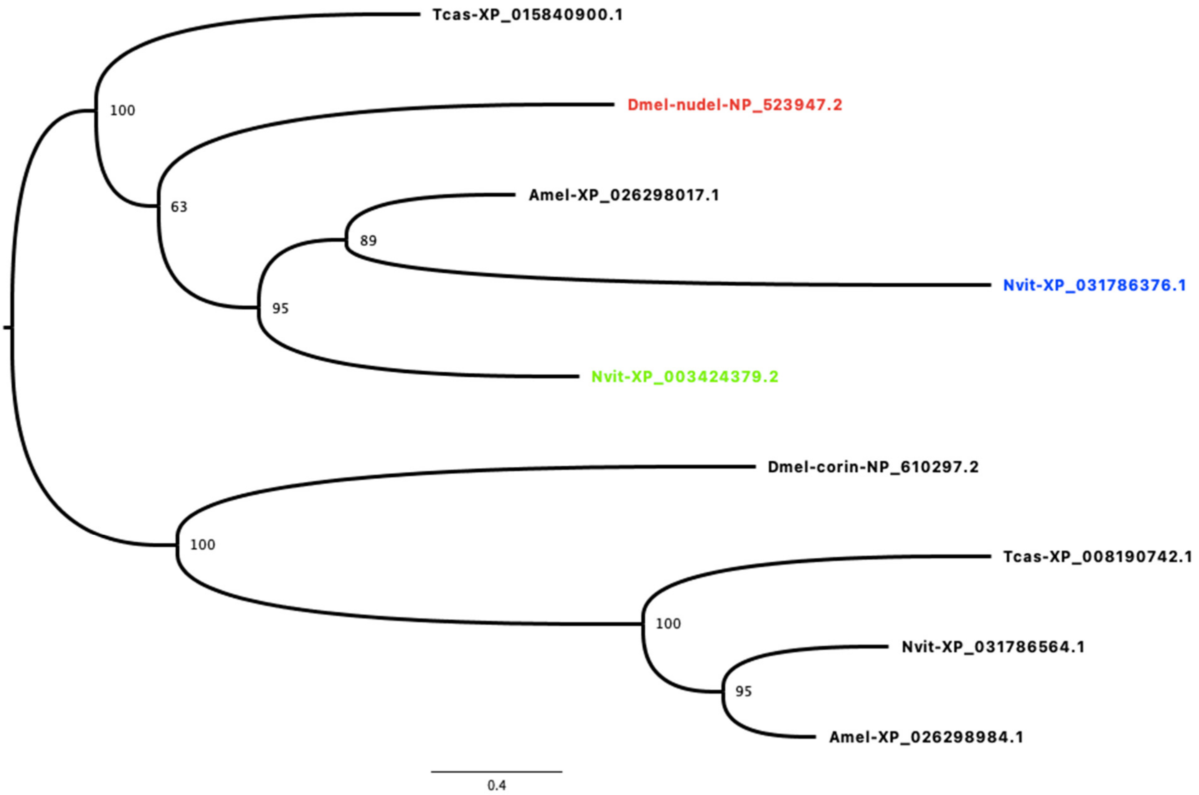

Supplementary Figure S3. Phylogenetic analysis of Nudel relatives. Likely Nasonia ortholog of D. melanogaster Nudel (red) examined in text in green. Potential new Nudel ortholog in blue. Node labels are bootstrap support.

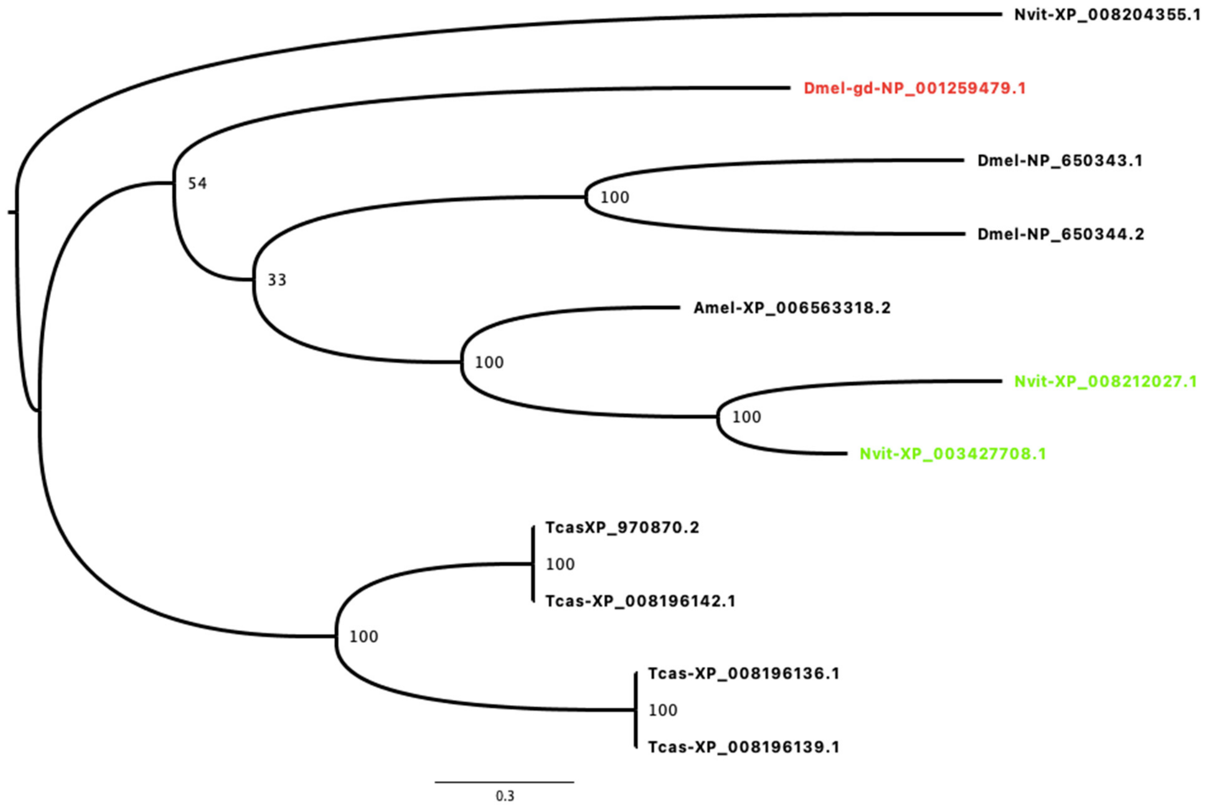

Supplementary Figure S4. Phylogenetic analysis of Gastrulation Defective (Gd). Likely Nasonia orthologs of D. melanogaster Gd (red) in green. Node labels are bootstrap support.

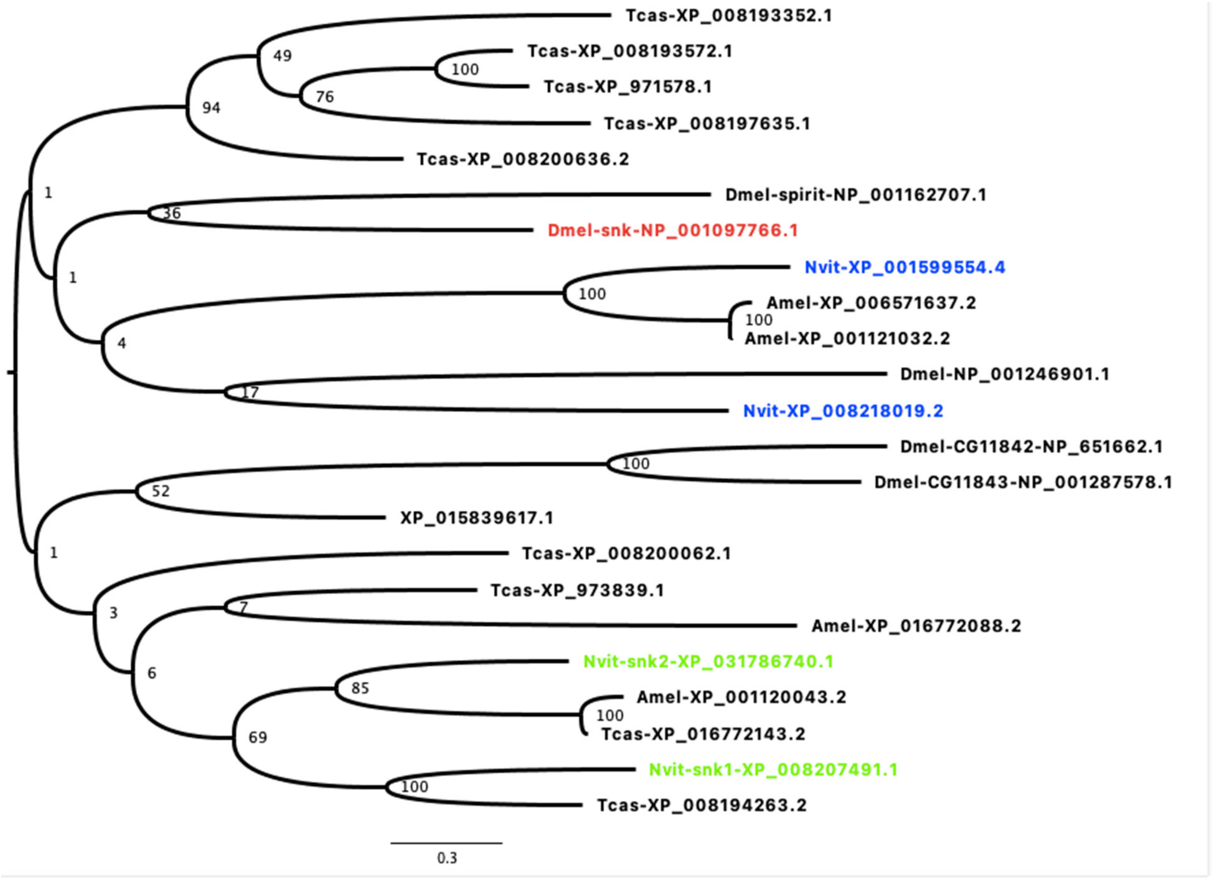

Supplementary Figure S5. Phylogenetic analysis of Snake (Snk). Potential Nasonia orthologs of D. melanogaster Snk (red) examined in the text in green. Potential additional orthologs in blue. Node labels are bootstrap support.

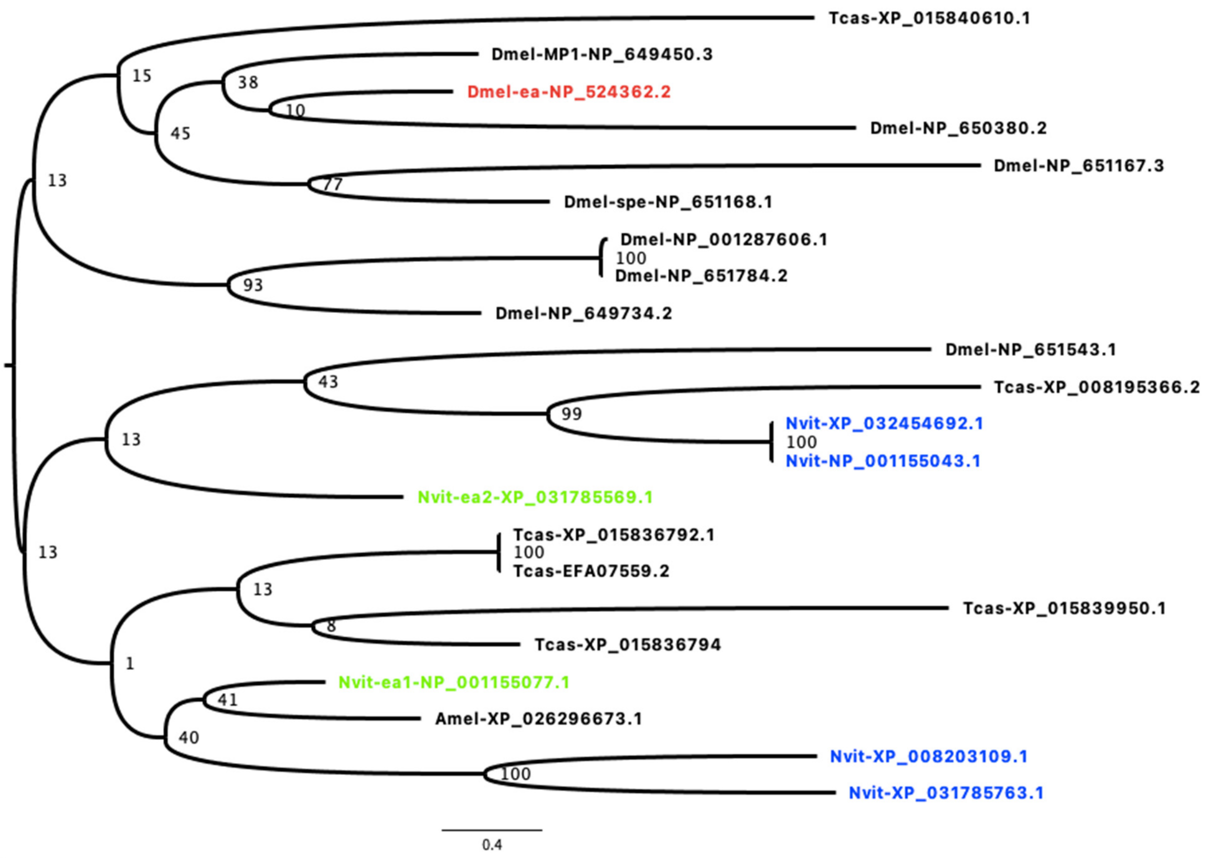

Supplementary Figure S6. Phylogenetic analysis of Easter (Ea). Potential *Nasonia* orthologs of D. melanogaster Ea (red) examined in the text in green. Potential additional orthologs in blue. Node labels are bootstrap support.

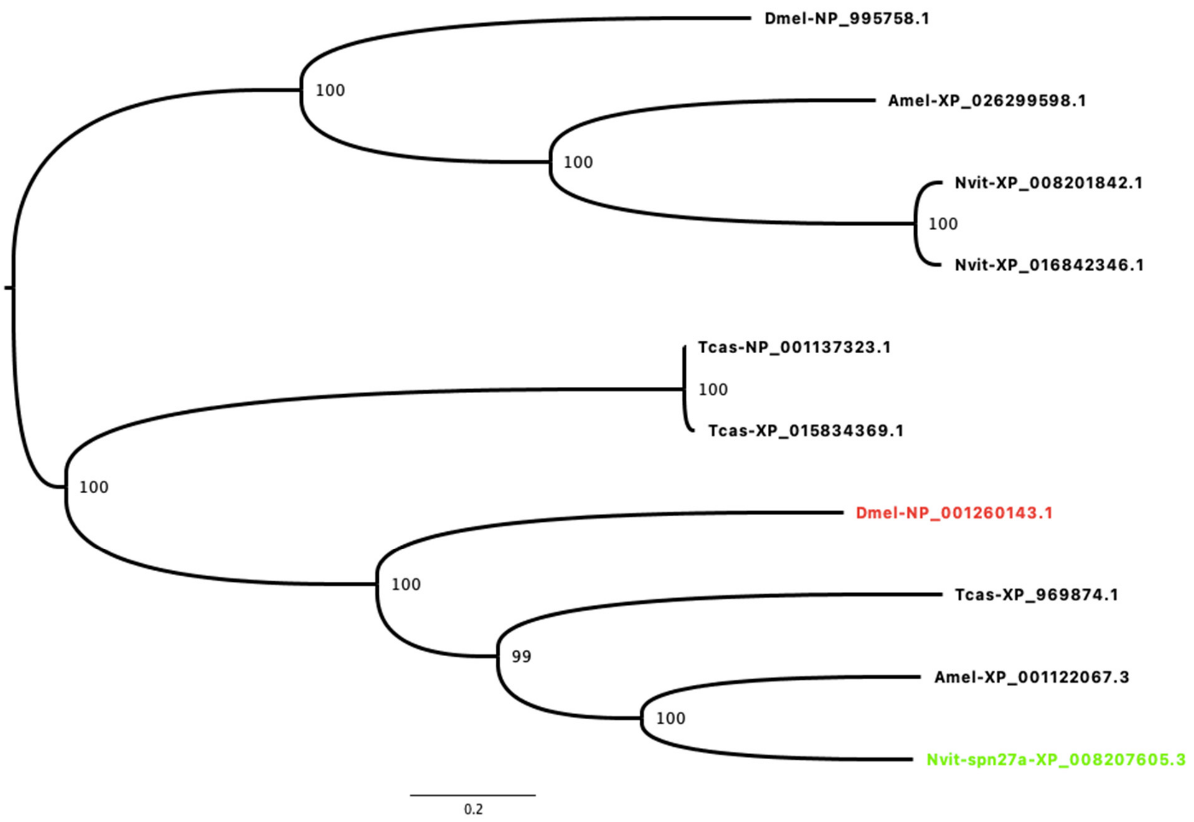

Supplementary Figure S7. Phylogenetic analysis of Serpin 27a (Spn 27a). Potential Nasonia orthologs of D. melanogaster Snake (red) examined in the text in green. Potential additional orthologs in blue. Node labels are bootstrap support.

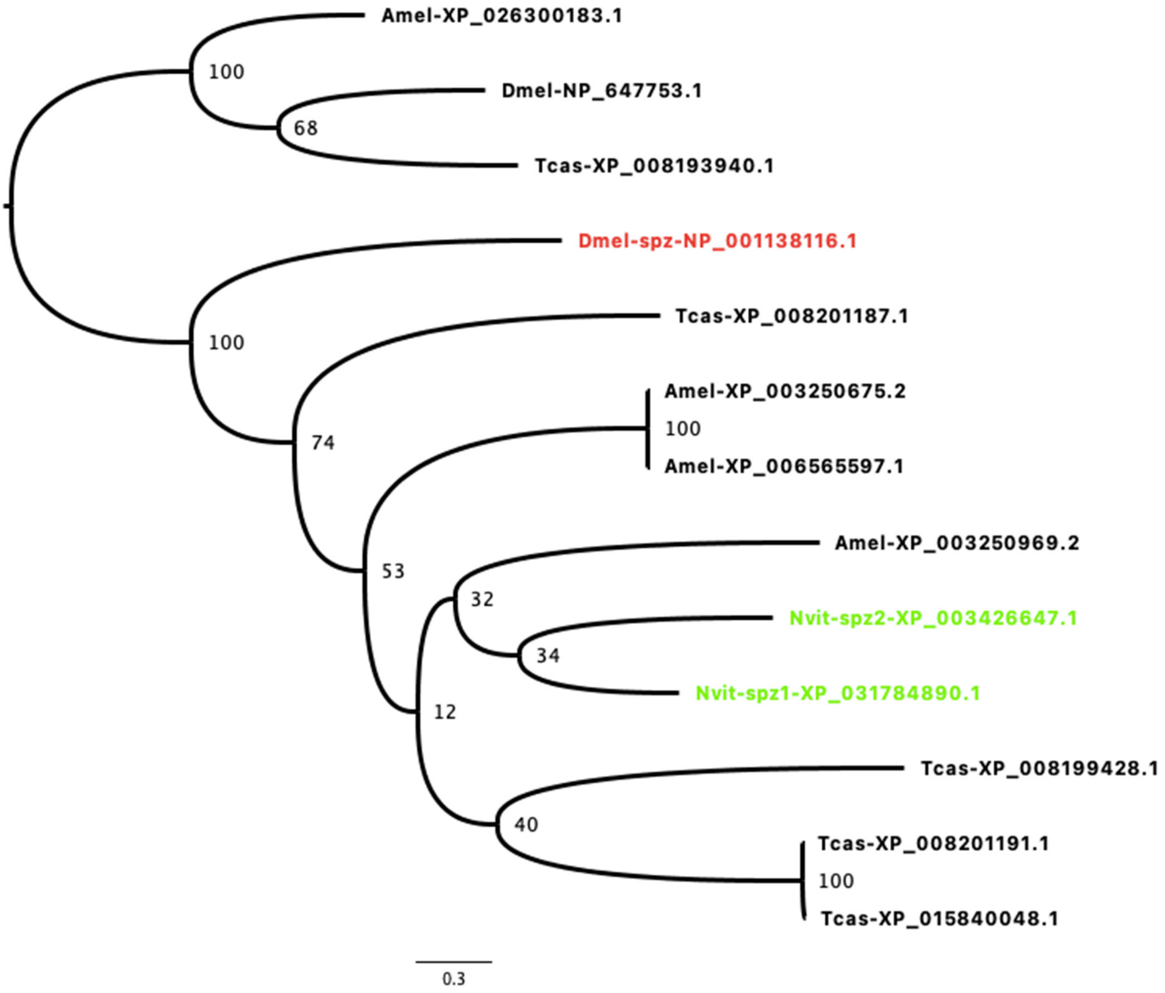

Supplementary Figure S8. Phylogenetic analysis of Spaetzle (Spz). Potential Nasonia orthologs of D. melanogaster Spz(red) examined in the text in green. Node labels are bootstrap support.

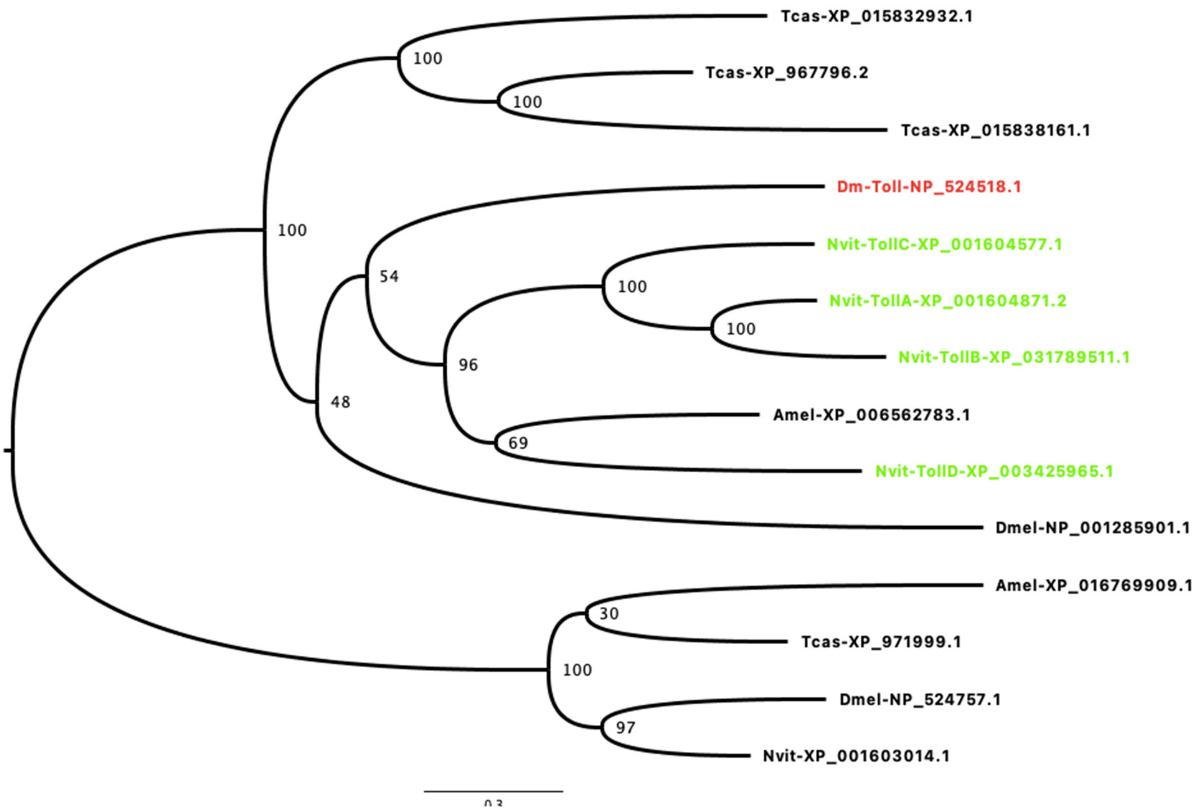

Supplementary Figure S9. Phylogenetic analysis of Toll. Potential Nasonia orthologs of D. melanogaster Toll (red) examined in the text in green. Node labels are bootstrap support.

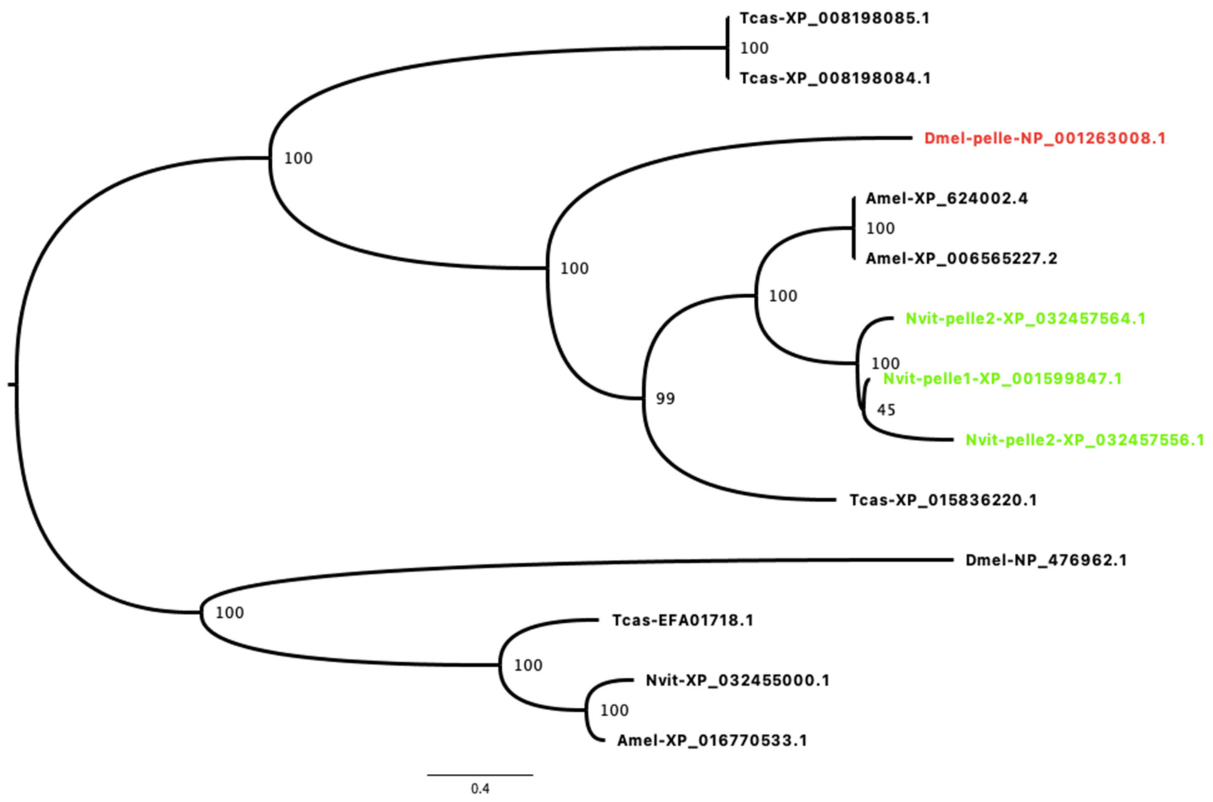

Supplementary Figure S10. Phylogenetic analysis of Pelle. Potential Nasonia orthologs of D. melanogaster Pelle (red) examined in the text in green. The most recent annotation split Nv-pelle2 between two protein predictions. Node labels are bootstrap support.

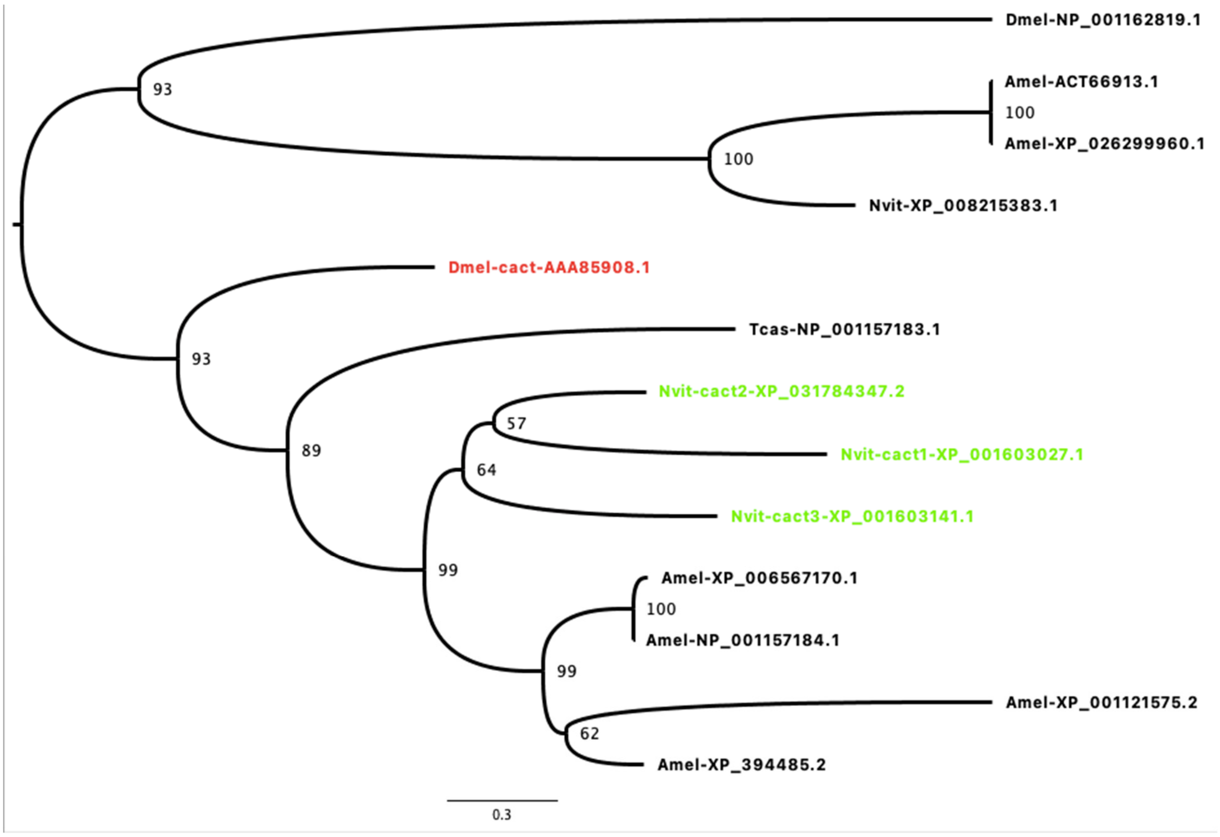

Supplementary Figure S11. Phylogenetic analysis of Cactus (cact). Potential Nasonia orthologs of D. melanogaster Cactus (red) examined in the text in green. Node labels are bootstrap support.

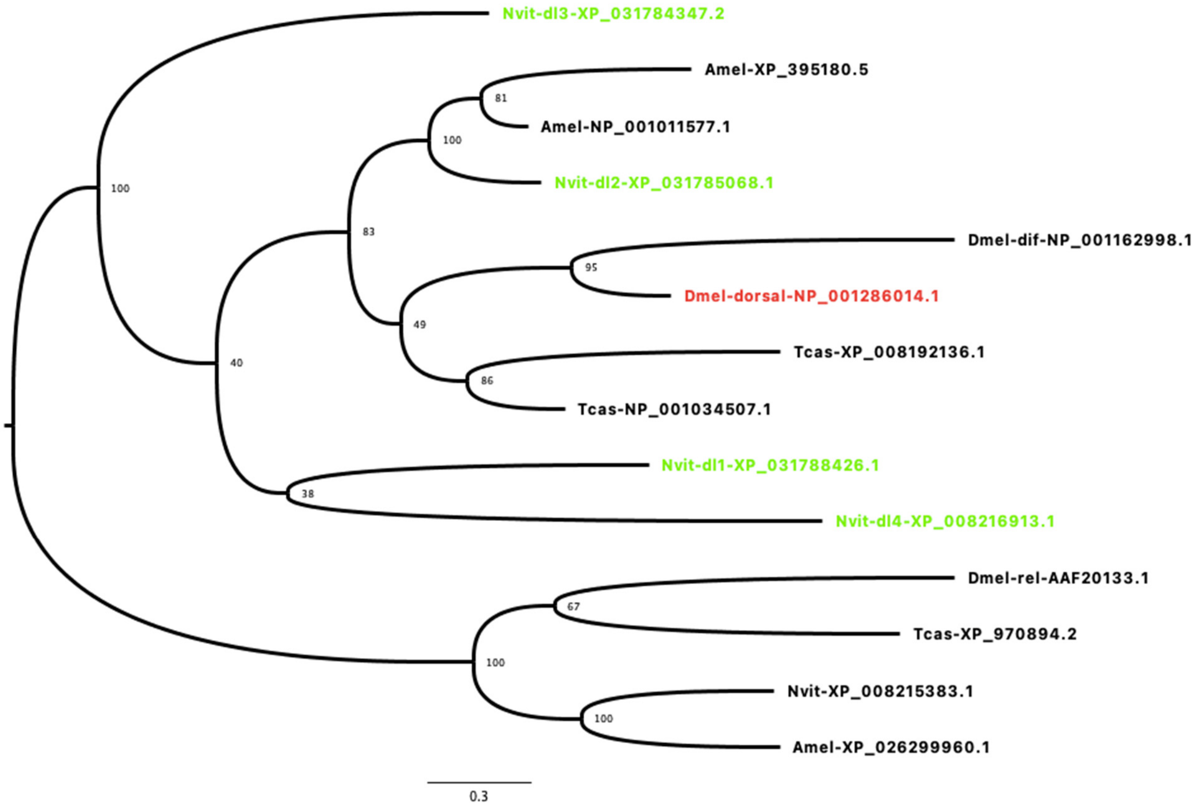

Supplementary Figure S12. Phylogenetic analysis of Dorsal (dl). Potential Nasonia orthologs of D. melanogaster Dl (red) examined in the text in green. Node labels are bootstrap support.
